# Food Marketing towards Children: Brand Logo Recognition, Food-Related Behavior and BMI among 3–13-Year-Olds in a South Indian Town

**DOI:** 10.1371/journal.pone.0047000

**Published:** 2012-10-17

**Authors:** Peter Ueda, Leilei Tong, Cristobal Viedma, Sujith J. Chandy, Gaetano Marrone, Anna Simon, Cecilia Stålsby Lundborg

**Affiliations:** 1 Karolinska Institutet, Stockholm, Sweden; 2 Stockholm School of Economics, Stockholm, Sweden; 3 Royal Institute of Technology, Stockholm, Sweden; 4 Christian Medical College, Vellore, India; 5 Division of Global Health (IHCAR), Karolinska Institutet, Stockholm, Sweden; University of Buea, Cameroon

## Abstract

**Objectives:**

To assess exposure to marketing of unhealthy food products and its relation to food related behavior and BMI in children aged 3–13, from different socioeconomic backgrounds in a south Indian town.

**Methods:**

Child-parent pairs (n = 306) were recruited at pediatric clinics. Exposure to food marketing was assessed by a digital logo recognition test. Children matched 18 logos of unhealthy food (high in fat/sugar/salt) featured in promotion material from the food industry to pictures of corresponding products. Children's nutritional knowledge, food preferences, purchase requests, eating behavior and socioeconomic characteristics were assessed by a digital game and parental questionnaires. Anthropometric measurements were recorded.

**Results:**

Recognition rates for the brand logos ranged from 30% to 80%. Logo recognition ability increased with age (p<0.001) and socioeconomic level (p<0.001 comparing children in the highest and lowest of three socioeconomic groups). Adjusted for gender, age and socioeconomic group, logo recognition was associated with higher BMI (p = 0.022) and nutritional knowledge (p<0.001) but not to unhealthy food preferences or purchase requests.

**Conclusions:**

Children from higher socioeconomic groups in the region had higher brand logo recognition ability and are possibly exposed to more food marketing. The study did not lend support to a link between exposure to marketing and poor eating behavior, distorted nutritional knowledge or increased purchase requests. The correlation between logo recognition and BMI warrants further investigation on food marketing towards children and its potential role in the increasing burden of non-communicable diseases in this part of India.

## Introduction

In the light of the ongoing epidemic of child obesity and non-communicable diseases globally, food marketing towards children has been increasingly recognized as an important health risk. Marketing of food high in sugar, fat or salt, aimed at children is pervasive worldwide [1] and evidence points at considerable effects on food consumption and related behavior [2], [3].

India experiences a growing epidemic of non-communicable diseases, attributable to a strong gene-environmental interaction [4] and lifestyle changes caused by modernization and urbanization. The incidence of coronary heart disease has increased fourfold in the past 40 years [5] and with its 50.8 million diabetics, India is today the country with the highest number of patients suffering from the condition [6]. Child overweight is reported to be common in urban areas [7] reaching up to 20% in some studies [8]. Although a larger problem for the urban upper and middle income groups [9], non-communicable diseases are increasing in low socioeconomic groups also [10].

Concurrently, the marketing landscape surrounding the children in India is changing rapidly. Eager to tap into the emerging Indian market, multinational as well as domestic food companies have intensified their marketing presence using a wide array of techniques [11], [12]. Reportedly, advertising spent on Indian television was US$ 2.4 billion in 2010, growing at a yearly rate of 24% [13]. Of the commercials shown during children's television programming, it is observed that a substantial share are for food, mostly promoting processed packaged foods, soft drinks, snacks and junk foods [14]. In addition, the increasing promotion efforts in-store, in print, at schools and through other media, let alone the advent of internet, have further diversified the marketing channels aimed at children and complicated the mapping of them [15].

In this study the extent of exposure to food marketing was assessed among children from different age and socioeconomic groups in a town in southern India. Marketing exposure was indicated by children's ability to recognize brand logos featured in promotional material of the food industry. Studies have found that food marketing shifts children's eating behavior towards a more unhealthy diet by affecting preferences [16], [17] and consumption choices [18]. Furthermore, children affected by advertisements influence parents in food purchase decisions to buy the advertised products; a behavior known as “pestering” or purchase requests [19]. In some cases, food marketing has also been shown to alter children's knowledge of food content and healthiness [20] by implying that advertised products are more healthy than they actually are. Thus, the relationship between logo recognition ability and eating behavior, food preferences and nutritional knowledge as well as behavior related to purchase requests was investigated. To assess marketing exposure in relation to child overweight, BMI of the children was measured.

Previous studies have shown that children as young as three years of age are able to recognize brand logos from various product categories [21]. From this age they also start to develop food choices [22]. We studied children from the age of 3 up to 13.

The objectives of the study were (i) to assess the ability of 3–13 year old children to recognize brand logos that are featured in food promotional campaigns and how this ability relates to age and socioeconomic status, (ii) to investigate food-related behavior - i.e. eating behaviors, food preferences, nutritional knowledge and purchase requests - and BMI in these children (iii) to investigate the relationship between individuals' logo recognition ability and their food-related behavior and BMI.

## Methods

### Study design and development of logo recognition instrument

This was a cross-sectional study, based on digital games and anthropometric measurements for children and a parental questionnaire. Brand logo recognition – as first developed by Fischer et al [21] – was used as a measure of exposure to marketing activities. The logo recognition was tested by assessing the child's ability to match a logo to a corresponding product category. Logos were chosen from a wide range of soft drink, junk food, snacks and sweets products from both Indian and international companies. (Table 1) The selection of logos was based on a review of frequently advertised food products during children's programming on TV and on community billboards in the region. For the test, we developed a picture-based application for a touchscreen tablet computer (Kendo M7) running Android 2.1.

**Table 1:** Brand logos, corresponding products and the number of children correctly recognizing the logos.

Brand	Product category	Correctly recognized n (%)
Boost	Chocolate drink/brown soft drink	217 (80)
Maaza	Orange soft drink	209 (77)
Lay's	Chips	197 (73)
Gems	Candy	192 (71)
Pepsi	Brown soft drink	189 (70)
Coca Cola	Brown soft drink	181 (67)
KitKat	Chocolate	161 (60)
Cadbury	Chocolate	154 (57)
20-20	Biscuits	153 (57)
Tiger	Biscuits	148 (55)
Rasna	Orange soft drink	128 (47)
KFC	Fried chicken/burgers	121 (45)
Mirinda	Orange soft drink	118 (44)
Centerfresh	Chewing gum	110 (41)
Domino's	Pizza	95 (35)
Oreo	Biscuits	91 (34)
Parle	Biscuits	89 (33)
McDonald's	Burgers	81 (30)

270 children completed the brand logo recognition test. The 12^th^ category was “noodles” and did not belong to any of the brand logos.

Test of children's food preferences and nutritional knowledge was fashioned after a scale developed in the U.S. by Calfas et al [23] and modified to suit an Indian setting. Ten matched pairs of food types - one healthy alternative and one unhealthy - were selected (Table 2). In this scale children's preferences are indicated by which food type in each pair they prefer and nutritional knowledge by which one they consider to be the healthy alternative. A picture-based touchscreen application was also developed for these tests.

**Table 2:** Pairs of food alternatives for the food preference and nutritional knowledge test.

Alternative 1 healthy	Alternative 2 unhealthy
Juice	Cola
Nuts	Chips
Mango	Candy
Fruits	Chocolate
Banana chips	Cookies
Korma rice	Pizza
Rice	French fries
Chapati	Burgers
Homemade food	Fast food
Milk	Chocolate drink

A questionnaire for parents collected information on children's birth date and gender. Parents' occupation, religion, monthly family income, education, house type and land ownership were also asked for.

Six questions related to purchase requests were prepared for parents to indicate how they perceive children's purchase request behavior.(Table 3) Answers were given on a four item Likert like scale consisting of “never”, “rarely”, “often”, or “always”. The child's consumption frequency of crisps, biscuits, sweets, deep fried food and soft drinks was reported by parents choosing from alternatives grouped as “once a day or more”, “once a week or more” or “less than once a week”. Questionnaires were available in English and Tamil.

**Table 3:** Children's eating behavior and purchase requests as reported by parents.

Eating behavior	≥once a day n (%)	≥once a week n (%)	<once a week n (%)
Chips (snacks)	109 (38)	129 (45)	51 (18)
Biscuits	235 (80)	45 (15)	13 (4)
Chocolate/candies	152 (52)	111 (38)	27(9)
Deep fried food (incl. home-made)	74 (26)	141 (49)	72 (25)
Soft drink	67 (24)	135 (48)	78 (28)

Purchase request behavior	Always n (%)	Often n (%)	Rarely n (%)	Never n (%)
Tries to influence in food purchase situations	80 (27)	85 (29)	89 (30)	39 (13)
Succeeds in influencing in food purchase situations	59 (20)	79 (27)	106 (36)	48 (16)
Conflicts when choosing food products	53 (18)	60 (21)	114 (39)	64 (22)
Shows independence and confidence when choosing food products	135 (46)	63 (22)	63 (22)	32 (11)
Influenced by advertisements on TV	66 (23)	69 (24)	82 (29)	70 (24)
Demand advertised brands/products	60 (21)	63 (22)	86 (30)	74 (26)

Given an average logo recognition score of 9 and standard deviation of 4, a sample size of 63 children concluding the brand logo test in each of the three socioeconomic groups was calculated to suffice to detect differences in scores of 2 or more between the groups (significance α = 0.05, power 80%).

### Study population

The study was conducted at the pediatric clinics of Christian Medical College Hospital in Vellore (CMC Vellore), Tamil Nadu in south India. Vellore and its surroundings have around 800 000 inhabitants, 500 000 of them living in the urban areas. Being one of India's largest pediatric health care providers with private and public units, the pediatric clinics at CMC Vellore has visitors - approximately 150–300 per day - from a wide span of socioeconomic groups in the region and beyond.

Children and their parents, visiting the hospital for various reasons, e.g. health checkups, vaccine boosts or accompanying patient siblings, were recruited from the waiting room of the clinic. Children matching the age criteria during the seven days of data gathering were approached. Children who were considered too sick to participate were not included. Children and parents speaking Tamil, Hindi or English were included. Parent consent and child assent was obtained for each child participating in the study.

### Data collection

Ethical approval was obtained from the institutional review board of the hospital prior to launching the data collection. The logo matching test and the preference and knowledge tests were first piloted and modified accordingly. Data collection was conducted during 7 days in April 2011 by PU, LT and CV with the assistance of translators. The test conductors used uniform procedures for recruiting and communicating with study participants.

Children completed the brand logo test and the preference and knowledge tests by touching objects appearing on the tablet screen. A pre-test was assigned in which the child was asked to match an object to a corresponding category picture (e.g. apple to fruits, monkey to animals) in order to assess the child's understanding of the test. Those who did not pass the pre-test were excluded from the logo matching quiz. The logo matching test constituted 12 pictures of products shown on the screen. One logo appeared at a time and the child was asked to touch the product that the logo represented. In total 18 logos were tested for.

After completion of the logo matching test, the preference and nutritional knowledge tests were conducted. Ten pairs of food or drink products appeared on the screen. The child was first told to touch the alternative he/she preferred. The same pairs then appeared on the screen again. This time the child was told to touch the alternative he/she thought was the healthiest. The children were uniformly asked “which one do you like?” and “which one is good for your body?”

Throughout the procedure the child was given praise regardless of the correctness of his/her answers. The pictures used in the tablet computer application were presented against a white background and selected to be of equal size and angles so as not to introduce a bias in children's choices. Test conductors, parents or translator were only allowed to explain the matching game and the preference/knowledge questions and were carefully instructed not to affect the child's answers.

The parental questionnaire was filled in by the parent in cases the parent could read English or Tamil. Translators assisted Hindi speaking participants and participants not being able to read. Weight and height of the child were measured using a digital weight scale and a measuring tape.

### Variables and statistical analysis

Brand logo score (ranging from 0 to 18) was defined as the number of correctly recognized brand logos by the child. Food preference score (0–10) was determined by the number of times the child preferred the healthier alternative in the matched pairs. The number of times the child identified the healthier alternative constituted the nutritional knowledge score (0–10). Purchase request score (1–4) was calculated as the average score from the questions on the four item Likert scale. (“Never” = 1, “Always” = 4) A higher number indicated a more frequent purchase request behavior. Eating behavior score (1–3) was the average score on the questions on how often the child consumed certain unhealthy products (“once a day or more” = 3, “once a week or more” = 2, “less than once a week” = 1). BMI was calculated according to the formula weight (kg)/height(m)^2^. Overweight was defined according to gender and age dependent cut-off points as proposed by Cole et al. [24]

Socioeconomic group (1–3) was decided using parents' educational level, family income, house type and land ownership. A score was given for each part and summed to a SES (socioeconomic status) score of maximum twelve. The subjects were grouped into three groups with cut-off points corresponding to approximately one third of the sample. A higher SES score indicated a higher SES. (Table 4)

**Table 4:** Distribution for gender, age, religion, family income and parents' education, land ownership and house type for the children participating in the study.

Gender	n (%)
Boys	177 (58)
Girls	127 (42)
**Age**	
3	22 (8)
4	29 (10)
5	30 (10)
6	31 (11)
7	32 (11)
8	34 (12)
9	34 (12)
10	31 (11)
11	21 (7)
12	18 (6)
13	11 (4)
**Religion**	
Hindu	233 (76)
Christian	33 (11)
Muslim	28 (9)
Unknown	12 (4)
**Family income (INR)** 1	
0–3500	79 (27)
3501–9000	76 (26)
9001–20000	74 (25)
>20000	67 (23)
**Mother's education**	
None	34 (11)
1–6 years	36 (12)
7–13 years	137 (45)
Graduate or higher	99 (32)
**Father's education**	
None	27 (9)
1–6 years	28 (9)
7–13 years	125 (41)
Graduate or higher	126 (41)
**Land ownership**	
Yes	191 (62)
No	115 (38)
**House type**	
Kutcha/Pucca^2^	173 (57)
Independent house/apartment	133 (43)

SES score was calculated from family income (0–3500 = 1, 3501–9000 = 2, 9001–20 000 = 3, >20 000 = 4) father's education (None = 0, 1–6 years = 1, 7–13 years = 2, Graduate or higher = 3), mother's education, land ownership (No = 0, Yes = 1) and house type (Kutcha/Pucca = 0, Independent house/apartment = 1).

1 1 USD = 44.58 Indian Rupees (www.oanda.com, 8 April 2011, accessed 20 April 2012).

2 Pucca is a house made of burnt bricks, stones, cement concrete, timber or ekra. Kutcha is a house made of material other than the mentioned above, such as un-burnt bricks, bamboos, mud, grass or reeds. (The Ministry of Statistics and Programme Implementation in India).

Data analysis was performed using a SPSS-13.0 statistical software package. Brand logo score, nutritional knowledge score and preference score were regarded as count variables. Non-parametric tests were employed to examine the bivariate relation between those variables and age (Spearman's correlation coefficient), gender (Mann-Whitney U-test) and SES group (Kruskal-Wallis test followed by Bonferroni-corrected Mann-Whitney U-tests comparing individual groups). Eating behavior score and purchase request score were continuous variables. Bivariate analyses were conducted for those variables and age (Pearson correlation coefficient), gender (t-test) and SES group (ANOVA followed by Bonferroni-corrected t-tests). SES group differences in age and BMI were examined with ANOVA followed by Bonferroni-corrected t-tests comparing individual groups. Differences in prevalence of overweight and gender by SES groups were tested using chi-square tests. To investigate how brand logo score related to food-related behavior, Poisson regression models with nutritional knowledge score and preference score as dependent variables and linear regression models with eating behavior score, purchase request score and BMI as dependent variables were specified. In each model, brand logo score, age, gender and SES group were independent variables. Furthermore, a Poisson regression model with brand logo score as dependent variable and age, gender and SES group as independent variables was used to examine the determinants of brand logo recognition. P-value <0.05 was considered significant in the final models.

## Results

### Population characteristics

Three-hundred and six parent-child pairs took part in the study. Two-hundred and fifty-eight children completed the logo test, the preference test and the knowledge test. Of the children who did not take part in the logo matching test but whose parents filled in the questionnaire, 14 were excluded as they did not pass the pre-test. Thirty-four did not complete their participation for other reasons, e.g. refusal by the child, language problems or time constraints.

Subjects ranged in age from 3 to 13 years and average age was 7.6 years (SD = 2.8). One-hundred and seventy-seven (58%) were boys. The majority of the children (233) were Hindu. Thirty-three were Christian, 28 Muslim and 12 belonged to other religions or did not report any religion. Large differences were observed between family incomes, ranging from 0 to 250 000 rupees (1 USD = 44.58 Indian Rupees (www.oanda.com, 8 April 2011, accessed 20 April 2012)) per month. Median value was 8000 rupees. Of the subjects' parents the majority of the mothers and fathers had more than eight years of education (77% and 82% respectively). Thirty-three percent of the mothers and 41% of the fathers had an educational record equivalent to a graduate degree or higher. Seventy-four of the children were categorized as belonging to the low socioeconomic (SES) group. One-hundred and twenty-five were placed in the middle and 97 in the high SES group. Age (p = 0.680) and gender (p = 0.582) of the children were equally distributed between the groups. Population characteristics are shown in table 4.

### Determinants of brand logo recognition ability

The average brand logo score was 9.8 (SD = 4.3) or 54% correct answers. No differences were found between genders (p = 0.400). Not surprisingly, higher age was associated with a higher brand logo score (Spearman's correlation coefficient = 0.496, p<0.001). Children in the high SES group scored significantly higher on the brand logo test compared to children from the low SES group (p<0.001). (Table 5)

**Table 5:** Comparison of purchase request score, eating behavior score, brand logo score, preference score, nutritional knowledge score, BMI and prevalence of overweight in children from different socioeconomic groups.

	Overall		Low SES group		Middle SES group		High SES group	
	Mean	Std. Dev.	Mean	Std. Dev.	Mean	Std. Dev.	Mean	Std. Dev.
**Purchase request score (1–4)**	2.6	0.68	2.4	.0.71	2.6	0.69	2.7*	0.61
**Eating behavior score (1–3)**	1.7	0.45	1.8	0.47	1.6**	0.43	1.7	0.43
**Brand logo score (0–18)**	9.8	4.3	8.5	4.0	9.8	4.3	10.7*	4.2
**Preference score (0–10)**	4.5	2.1	4.5	1.9	4.5	2.0	4.5	2.2
**Nutritional knowledge score (0–10)**	7.4	2.1	6.9	2.1	7.2	2.2	8.1*	1.8
**BMI**	15.4	3.1	14.4	1.9	15.3	3.0	16.1*	3.8
**Percentage overweight**	11%	-	2%	-	11%	-	19%*	-

* = p<0.05/3 (Bonferroni correction) when comparing low and high SES group.

** p<0.05/3 (Bonferroni correction) when comparing low and middle SES group.

Mean recognition rates for all logos are shown in table 1. Boost (80%), Maaza (77%), Lays (73%), Gems (71%), Pepsi (70%) and Coca Cola (67%) had the highest recognition rates among the brand logos. Random guessing alone would yield a recognition rate of 8.3%, corresponding to one out of twelve possible answers.

### Nutritional knowledge, preferences, purchase requests and eating behavior

Average nutritional knowledge score when specifying which foods were “healthy” or “unhealthy” was 7.4 (SD = 2.1). Nutritional knowledge score increased with age (Spearman's correlation coefficient = 0.405, p<0.001) and was not correlated to gender (p = 0.21). Children's preferences were more oriented towards the unhealthy options (average score = 4.5 (SD = 2.1)). Preference score did not differ between genders (p = 0.33). Although not significant, preferences tended to become more unhealthy as age increased (Spearman's correlation coefficient = 0.103, p<0.095) Children from the high SES group received higher score on the nutritional knowledge test compared to the low (p<0.001) whereas preferences did not differ between the SES groups (p = 0.92). (Table 5)

According to the parental questionnaires, purchase request behavior was common in the study population. Parents stated on the four item Likert scale that their children “always” (27% of responding parents) or “often” (29%) tried to influence them in purchase situations, succeeded in doing so (“always” 20%, “often” 27%), showed independence and confidence when choosing food products (“always” 46%, “often” 22%) and demanded specific brands and products that they had seen advertised (“always” 21%, “often” 22%). (Table 3) Purchase request behavior was not significantly associated to age (p = 0.251) or gender (p = 0.205). High SES children had a more frequent reported purchase request behavior (p = 0.003) compared to the low SES group. (Table 5)

High consumption of snacks, sugared sweets and soft drinks was reported. A large proportion of the children consumed biscuits (80%), chips (38%), chocolates/sweets (52%) or soft drinks (24%) at least once per day. (Table 3) Eating behavior score was associated to age (Pearson correlation coefficient = 0.118 p = 0.047) but not to gender (p = 0.818). The low SES group had higher eating behavior score compared to the middle SES group (p = 0.002). (Table 5)

In a linear regression analysis adjusted for SES group, age and gender, no correlations were found between the ability to recognize the brand logos and eating behavior (p = 0.113) or purchase request behavior (p = 0.140). In Poisson models adjusted for the above mentioned variables, there was a positive relationship between brand logo recognition and nutritional knowledge (β = 0.034 p<0.001) but no correlation between brand logo recognition and preferences (p = 0.453). (Table 6)

**Table 6:** Relation between brand logo recognition and nutritional knowledge score, preference score, purchase request score, eating behavior score and BMI (adjusted for age, gender and SES group (1–3)) and determinants for brand logo score.

Dependent variable	Brand logo score	Age	Female gender	Middle SES group (2)	High SES group (3)
**Brand logo score** 1	-	0.083*(0.068 to 0.098)	−0.048(−0.130 to0.034)	0.135*(0.028 to 0.241)	0.265*(0.155 to 0.374)
**Nutritional knowledge score** 1	0.034*(0.020 to 0.047)	0.017(−0.004 to 0.037)	−0.036(−0.131 to 0.0590)	0.009(−0.132 to 0.113)	0.086(−0.044 to 0.215)
**Preference score** 1	0.006(−0.010 to 0.023)	0.014(−0.012 to 0.040)	0.073(−0.048 to 0.194)	−0.018(−0.170 to 0.135)	−0.017(−0.182 to 0.149)
**Purchase request score** 2	−0.017(−0.041 to 0.006)	−0.002(−0.039 to 0.035)	−0.095(−0.264 to 0.074)	0.197(−0.014 to 0.409)	0.405*(0.177 to 0.633)
**Eating behavior score** 2	0.013(−0.003 to 0.028)	0.014(−0.011 to 0.038)	0.008(−0.105 to 0.121)	−0.253*(−0.395 to −0.111)	−0.180*(−0.333 to −0.027)
**BMI** 2	0.128*(0.018 to 0.238)	0.218*(0.044 to 0.393)	−0.648(−1.452 to 0.156)	0.851(−0.155 to 1.857)	1.716*(0.634 to 2.798)

* = p<0.05.

1 Poisson regression.

2 Linear regression.

The parameters of Poisson regression models can be interpreted as difference in the logs of expected counts for a one unit increase in the predictor variable, given that the other predictor variables in the model are held constant. The parameters of linear regression models can be interpreted as difference in the expected values of the dependent variable for a one unit increase in the predictor variable, given that the other predictor variables in the model are held constant.

### BMI and its determinants

Children in the lowest SES group had a lower BMI compared to children the high SES group (p<0.001). 2% of the children in the low SES group were overweight compared to 19% in the high SES group. The difference was significant (p<0.001). (Table 5)

Brand logo score was associated to a higher BMI (β = 0.12, p = 0.022) in a linear regression model with brand logo score, gender, age and SES group as independent variables. (Table 6)

## Discussion

This is to our knowledge the first study assessing exposure to food marketing – indicated by brand logo recognition – and food related behavior and BMI among children in India. Among 3–13 year old children, we found recognition rates ranging from 30% to 80% for many brands of products containing large amounts of sugar, salt and fat. The brand recognition increased with age and was higher in the high socioeconomic group compared to the low. These findings imply that children in this study are exposed to marketing of foods and drinks of which high consumption leads to adverse health effects. Seemingly, the marketing messages also reach out to different socioeconomic groups.

Consistent with previous reports [25], the reported purchase request behavior indicates that children in this study influence their parents in purchase decisions. Furthermore, a large proportion of the children were reported to consume snacks, biscuits, sweets or soft drinks at least once per day. Such dietary patterns have been shown to predispose for obesity and related health problems [26], [27] Given the accelerating epidemic of non-communicable diseases in India and the increasing availability – both in terms of distribution channels and purchasing power – of fatty and sugary foods and drinks, the exposure to marketing, the dietary habits and the purchase request behavior as depicted in this study could warrant consideration from a public health point of view.

There was a significant association between logo recognition and BMI. Naturally, this finding is not tantamount to a causal relationship between exposure to marketing and higher BMI. It is plausible that children having a lifestyle associated to a high BMI also have a high exposure to marketing through television watching and consumption of unhealthy branded foods. However, this correlation implies that marketing exposure and brand awareness could be part of a lifestyle possibly related to risk for overweight and subsequent non-communicable disease in the region.

Just as a previous study from the UK using a similar design [28], our study did not demonstrate a sizeable relationship between logo recognition and the children's reported eating behavior and preferences. Purchase request behavior was also not associated to brand logo recognition. Thus, the study did not provide evidence for such correlations in this Indian setting. Nutritional knowledge, in this study defined as the ability to indicate the healthy option from pairs of food products, was positively associated to logo recognition; a finding that tell against marketing effects on nutritional knowledge, at least on a general level as tested in this study. Rather, the correlation could possibly be explained by children's ability to take in information from their environment.

Admittedly, to gauge effects of marketing on children is difficult. Logo recognition is one way in which exposure to marketing can be quantified but entails a number of problems. Firstly, there are potential confounders such as the educational background of the children. It is possible that children with better education possibilities scored higher on both the brand logo and the nutritional knowledge test, as their ability to grasp concepts and learn the test procedure was higher than their less privileged peers. However, measures were taken to minimize such confounding effects. All tests constituted solely of pictures on a digital device that was navigated by touching the screen and did not involve reading or writing. The picture based sampling solution also helped mitigate the language problems that are inevitable in linguistically dispersed India. Additionally, all children had to pass a training version of the matching game before undertaking the logo test. Those who did not pass were excluded. Exclusion of children with the lowest ability to rapidly understand the study's logo matching procedure, however, risks to have introduced a bias into the study population, e.g. with regard to age and socioeconomic background.

It has to be acknowledged that the study site may not have been optimal for the children's test performances. Children visiting the hospital ward - either as patients themselves or accompanying their patient siblings - could be more stressed than usual thus decreasing their accuracy when tested in this study.

On the other hand, the study site provided us with the possibility to obtain information from both the parent and the child at the same time. It also gave access to a study population with a wide range of family incomes, parental education and other socioeconomic factors.

There is an apparent arbitrariness to the selection of logos and product categories. Focusing on products generally considered as components of an unhealthy diet, we tried to include a wide range of brands – both domestic and international - which we expected to have high recognition rates in the study region. As seen in the logo test, not all brands were widely recognized. In particular, multinational fast food chains such as McDonald's (30%) and Dominos (35%) received low proportions of correct answers. This was not entirely unexpected as these fast food chains are not present in Vellore. Reportedly, these multinationals are also not advertising frequently on local TV in the region. Although they were still recognized by one in three children, this could explain their relatively low recognition rates compared to other brands. It is thus of note that the logo test was not composed as to seek the highest possible brand recognition rate by the study population. There were certainly brands with potentially higher recognition that were omitted.

Generalizing the findings to the region and other parts of India is not strictly possible given the single geographic location, the study population being children visiting a pediatric clinic and the exclusion of children and parents not speaking Tamil, English or Hindi. However, children from a wide range of educational backgrounds and family incomes participated in the study and constituted a study population with a socioeconomic spread comparable to data in population censuses from the region [29].

In conclusion, this study investigated the current situation regarding marketing of foods and drinks in a primarily urban setting in southern India by assessing the degree of marketing exposure and food-related behavior and BMI in a population of children from different age and socioeconomic groups. Recognition rates for the 18 logos tested in the study ranged from 30% to 80%. Furthermore, parents reported poor dietary habits and frequent purchase request behavior among their children. The study, however, did not provide evidence for a link between exposure to marketing and poor dietary habits, distorted nutritional knowledge or increased purchase request behavior. A correlation was found between brand logo recognition and higher BMI independent of socioeconomic group, age and gender. Future studies assessing the effects of marketing exposure on risk for child overweight could thus be warranted to provide information for decisions on marketing regulations in the region. Further examination of this topic is needed to elucidate the potential role of food marketing in the promotion of healthy lifestyles from early age and in the combat against non-communicable diseases in modern India.
